# Effect of mycorrhizae on phosphate fertilization efficiency and maize growth under field conditions

**DOI:** 10.1038/s41598-023-30128-7

**Published:** 2023-03-02

**Authors:** Fernando de Souza Buzo, Lucas Martins Garé, Nayara Fernanda Siviero Garcia, Maura Santos Reis de Andrade Silva, Juliana Trindade Martins, Pedro Henrique Giova da Silva, Flávia Constantino Meireles, Leticia Zylmennith de Souza Sales, Amaia Nogales, Everlon Cid Rigobelo, Orivaldo Arf

**Affiliations:** 1 Department of Plant Science, Food Technology and Socio-Economics, Faculty of Engineering of Ilha Solteira/UNESP, Ilha Solteira, SP Brazil; 2 Department of Plant Production, Faculty of Agriculture and Veterinary Sciences/UNESP, Jaboticabal, SP Brazil; 3 Department of Agroecology, Aarhus University, Blichers Allé 20, 8830 Tjele, Denmark; 4 Linking Landscape, Environment, Agriculture and Food (LEAF), Superior Institute of Agronomy (ISA), University of Lisboa, Lisboa, Portugal

**Keywords:** Arbuscular mycorrhiza, Field trials, Plant sciences

## Abstract

Phosphorus (P) is a plant macronutrient that is indispensable for maize (*Zea mays* L.) production. However, P is difficult to manage in weathered soils, and its fertilization practice has low efficiency because it becomes unavailable for absorption by plant roots. Symbiosis of plants with arbuscular mycorrhizal fungi increases plant growth and enhances P uptake from the soil that is not directly available to the roots. Thus, the objective of this study was to determine how inoculation with *Rhizophagus intraradices* and phosphate fertilization interacts and influences the development and productivity of second-crop maize. The experiment was conducted in Selvíria, Mato Grosso do Sul, Brazil, in 2019 and 2020, both in a Typic Haplorthox. A randomized block design in subdivided plots was used for the phosphate application during crop sowing (0, 25, 50, 75, and 100% concentrations of the recommended level), and the secondary treatments were the doses of mycorrhizal inoculant (0, 60, 120 and 180 g ha^−1^) applied to the seed using a dry powder inoculant containing 20,800 infectious propagules per gram of the arbuscular mycorrhizal fungus *R. intraradices*. Only in the first year of the experiment, inoculation and phosphate fertilization promoted benefits to the maize crop, indicating potential to increase yield.

## Introduction

Maize is considered as one the most important cereal crop worldwide given its relevance to animal and human nutrition as well as industrial processing^1^. In 2019, 197.20 million hectares were cultivated with maize globally, resulting in a production of 1.15 billion tons. This figure represents 27.23% of the world's area cultivated with cereals and 38.55% of cereal grains produced worldwide in the same year^2^.

The requirement of phosphorus (P) for maize is less compared to those of nitrogen (N) and potassium (K), but the amounts applied in Brazil are extremely high because of the low absorption of P by the roots^3^. This application is a consequence of the adsorption of P by iron and aluminum oxyhydroxides in the soil, making it unavailable for absorption by the roots^4–6^.

The plant kingdom has developed several strategies to fix P in the soil, including symbiosis with arbuscular mycorrhizal fungi (AMF). These fungi colonize plant roots and develop hyphae that diffuse into the soil, increasing the volume of soil explored by the plant and the uptake of water and nutrients, especially P^5,7,8^.

However, excessive P concentrations in the soil can inhibit mycorrhizal establishment and root colonization by AMF^7,9,10^, potentially leading to a decrease in the mycorrhizal growth response^11^. Therefore, to obtain the maximum gain from phosphate fertilization and mycorrhizal inoculation, the quantity of both components needs to be adjusted for each soil and crop type.

Although the benefits of mycorrhizal symbiosis are known, large-scale agricultural use of AMF-based inoculant is limited, since the processes involve high costs and do not work for every species of AMF^12^. Thus, the production cost of the inoculant can equal the cost of the phosphate fertilizer^13^ and also, the isolates produced tend to be uncompetitive with the native species on the soil^14^. For this reason, the use of AMF inoculants in large scale agriculture is still scarce, and most of the research conducted is done under laboratory and greenhouse conditions. Siqueira et al.^15^, presented a table with Brazilian studies of mycorrhizae and of the 50 listed, only 2 were conducted under field conditions. Similarly, Lekberg and Koide^16^ verified in a meta-analysis that the benefits of AMF for plants are smaller in field experiments than in greenhouse conditions.

The aim of this work was to test if, under field conditions, inoculation of maize with the fungus *Rhizophagus intraradices* could improve the development and yield of the crop, and if there is an interaction between the dose of inoculant and the dose of P_2_O_5_ applied, which would increase the efficiency of the application of a follow-up fertilizer.

## Methods

### Location of the experiment

The research was conducted in 2019 and 2020 in an experimental area located in the municipality of Selvíria, Mato Grosso do Sul, Brazil (51° 24′ 11.34" W, 20° 20′ 35.32" S), with an average altitude of 358 m. This is an area of the Cerrado region, that has been used for agricultural purposes for more than 20 years. In the two years of cultivation, different areas were used, but with the same characteristics and management, including the no-till system in the implementation phase, so that the experiment was the second and fourth agricultural crop in the area (completing 2 years since the beginning of the no-till system). In both years, millet (*Pennisetum glaucum*) was grown as a cover crop before maize.

The climate of the region is of type Aw (Köppen classification) and has an average annual rainfall of 1322 mm and average annual temperature of 23 °C^17^. The average temperature, rainfall, relative humidity, and insolation during the experiment are described in Supplementary Fig. 1.

The soil was classified as Typic Haplorthox (Oxisol) according to the Soil Survey Staff^18^. After collecting and analyzing the soil in the 0.00–0.20 m layer, the following chemical attributes were obtained for 2019: 16 mg dm^−3^ P (resin), 6 mg dm^−3^ S–SO_4_; 21 g dm^−3^ organic matter; pH 5.2 (CaCl_2_); 1.8, 28.0, 18.0, and 31.0 mmolc dm^−3^ K, Ca, Mg, and H + Al, respectively; 3.6, 21.0, 23.4, and 0.9 mg dm^−3^ Cu, Fe, Mn, and Zn (DTPA), respectively; 0.24 mg dm^−3^ B (hot water) and 61% base saturation. For 2020, the following chemical attributes were obtained: 25 mg dm^−3^ P (resin), 3 mg dm^−3^ S-SO_4_; 18 g dm^−3^ organic matter; pH 5.0 (CaCl_2_); 0.7, 19.0, 16.0, and 31.0 mmolc dm^−3^ K, Ca, Mg, and H + Al, respectively; 1.6, 25.0, 11.1, and 0.6 mg dm^−3^ Cu, Fe, Mn, and Zn (DTPA), respectively; 0.27 mg dm^−3^ B (hot water) and 54% base saturation.

In addition, a physical analysis of the soil was performed regarding its texture, and the following data was obtained: 38,2% sand, 10.3% silt and 51.5% clay for 2019 and 64.5% sand, 5.7% silt and 29.9% clay for 2020. To better understand the dynamics of phosphorus and its adsorption capacity in the soil of the two cultivation areas, the determination of the remaining P according to Alvarez et al.^19^ was also performed and the results were 25 mg L^−1^ in 2019 and 37 mg L^−1^ in 2020.

### Biological material

The maize (*Zea mays* L.), simple hybrid AG 7098^®^ was used in the experiment during 2019, whereas a coded experimental simple hybrid (unreleased) from the Bayer company was used during 2020. In the first year, seeds had been industrially treated with 2.0 g metalaxyl, 15.0 g thiabendazole, 2.5 g fludioxonil, 0.2 g deltamethrin, 0.8 g pyrimiphos methyl, per 100 kg seeds; whereas in the second year, untreated seeds were used.

The commercial dry powder inoculant Rootela BR^®^ was used for seed inoculation, as it was the only commercial inoculant available for grain cultivation in Brazil at the time of the experiment. The product is manufactured by Ground-Work BioAg (Israel)^20^ and imported and marketed in Brazil by NovaTero. It contains 20,800 infectious propagules per gram of the arbuscular mycorrhizal fungus *Rhizophagus intraradices* (Schenck and Smith) in a mixture of 82% vermiculite, 6% clay, and 12% unidentified particles^21^.

### Experimental design and treatments

The experimental design used was a randomized block design in subdivided plots, with four repetitions. The P_2_O_5_ was applied in the plots and the inoculant was applied in the subplots. The amounts of P used corresponded to 0, 25, 50, 75 and 100% of the recommended doses for the maize crop in the area, according to Raij and Cantarella^22^. This resulted in levels of 0, 30, 60, 90 and 120 kg ha^−1^ of P_2_O_5_ for the year 2019 and 0, 15, 30, 45 and 60 kg ha^−1^ of P_2_O_5_ for the year 2020. The amounts of *R. intraradices* inoculum used were 0, 60, 120 and 180 g ha^−1^ (Supplementary Fig. 2). Each plot received a dose of the corresponding phosphate fertilizer and featured four subplots with four rows, each 7 m long, and an inter-row spacing of 0.85 m. For the analyses, the two central rows were considered as useful areas, disregarding the distance of 0.5 m from the ends.

### Installation and conduct of the experiment

Sowing was performed on 03/15/2019 in the first year of the experiment and on 03/10/2020 in the second year of the experiment, after straw management with a field shredder. Seed fertilization was 60 kg ha^−1^ of K_2_O and 45 kg ha^−1^ of N in the first year and 60 kg ha^−1^ of K_2_O and 33 kg ha^−1^ of N in the second year of cultivation, according to the recommendation for the area^14^. The N was supplied with urea and MAP (except for the 0 dose of P_2_O_5_, in which all N was supplied via urea) and K_2_O was supplied with KCl. The P_2_O_5_ fertilization was provided using monoammonium phosphate (MAP; 52% P_2_O_5_ and 11% N) as the source. The balanced application of these fertilizers was done with a no-till seed drill, supplied only with fertilizers, with fertilizer applied in the seed furrows created by the drill. The fertilizer deposition was regulated to an average depth of 8 cm. Each dose of P_2_O_5_ was adjusted and applied to the corresponding plots. Since MAP has N in its composition, it was necessary to adjust the urea dose in each plot so that the N content delivered to each plot was the same in all treatments.”

Inoculation was carried out before sowing by mixing the seeds, the amount of inoculant needed for each treatment and a sugar solution (10%) in the proportion of 300 mL 50 kg^−1^ of seeds to improve the adherence of the inoculant to the seeds. After mixing, the inoculated seeds were dried in the shade and sowing was performed. Sowing was done with a manual sowing machine, placing the seeds in pits every 0.20 m in the sowing lines, in order to obtain the recommended plant population (57,000 plants ha^−1^). All experimental blocks were sown simultaneously, and each block was sown by the same person. After sowing, the area was irrigated with 15 mm of water.

During the entire crop cycle, the water supply to meet the water demand of maize was supplied by a fixed sprinkler irrigation system with an average rainfall of 15 mm h^−1^ in 2019 and a central pivot with an average rainfall of 13 mm h^−1^ in 2020.Crop emergence occurred on 03/20/2019 in the first year and on 03/15/2020 in the second year. In the first year, cover crop nitrogen fertilization was performed with a supply of 60 kg ha^−1^ N at V_4_ and 60 kg ha^−1^ N at V_8_. In the second year of the experiment, 50 kg ha^−1^ of N at V_4_ and 60 kg ha^−1^ of N at V_8_ were applied. In both years of the experiment, only urea was used as a source of N in the cover crop, this was done by applying urea next to each row of plants. Phytosanitary and weed management was done according to crop recommendations and the needs of the studied area.

Phytosanitary and weed management performed in both years of the experiment were recommended for the crop and according to the demand of the area.

Female flowering of the crop occurred 50 and 45 days after emergence (DAE) in 2019 and 2020, respectively. The harvest was performed manually after grain maturation (115 DAE in 2019 and 107 DAE in 2020), and the ears were collected from two central rows (5 m each) in each plot.

### Evaluations performed

At the time of female flowering the following evaluations were performed:

- Leaf chlorophyll index (LCI): It was determined by taking readings with a portable chlorophyll meter (ChlorophiLOG^®^, model CFL 1030) in the middle third of the leaf laminae located opposite and below the main corncob of five plants per plot.
- Plant dry mass: Six consecutive plants were cut close to the ground and weighed, and the moisture content of each sample was estimated to calculate the dry mass corresponding to each plot. Data were converted to g plant^−1^.
- Percentage of root colonization by mycorrhizal fungi: Thin roots were collected from three plants at depths of 0.00–0.20 m, washed, and stored in 70% alcohol. Later, they were clarified and stained with trypan blue in 0.05% lactoglycerol^23^. Using an optical microscope (40 ×), 100 small root fragments were observed to verify the presence or absence of AMF structures, and the percentage of root colonization was determined^24^.
- Counting of mycorrhizal fungal spores: A composite soil sample was collected from the 0.00–0.20 cm layer of each plot, air-dried, and sieved. Then, a 50 mL soil sample from each plot and spores were separated using the decantation method and wet sieving. The material retained on the 0.053 mm sieve was centrifuged at 3000 rpm for 3 min. The supernatant was discarded, and a 50% sucrose solution was added to the tubes and centrifuged again at 2000 rpm for 2 min. The supernatant was poured into a plate with concentric rings to perform spore counts under an optical microscope (4x)^25^.

At the physiological maturity of maize (R_6_), the following evaluations were performed with 10 plants gathered from the useful area of the plots:

(e) Plant height: Distance from ground level to the apex of the maize stalk was measured using a graduated ruler.
(f) Height of corncob insertion: Distance from the ground level to the insertion of the main corncob was measured using a graduated ruler.
(g) Stalk basal diameter: It was measured using a digital pachymeter, and the second internode of the plant was used as the base.

After harvest, ten ears per plot were separated to perform the following evaluations:

(h) Average corncob length and diameter: The length of the dehusked corncob was determined using a graduated ruler and the diameter of the central third of each corncob, without the removal of the grains, using a pachymeter.
(i) Average number of grains per row and number of rows of corncobs: The number of rows and the number of grains in a representative row of each of the ten corncobs were counted to calculate the averages.
(j) Mass of the dehusked corncob, cob, and grain per corncob: The dehusked corncobs were weighed (mass of dehusked corncob) and then the grains were removed to determine the mass of grains and cobs.

After the harvest and the analysis of the previous variables, mechanized threshing of the corncobs was performed, and the following analyses were made:

(k) Mass of 1000 grains: counting and weighing 250 grains of each plot and, with the determination of the moisture content of each plot, the mass of 1000 grains was estimated with a moisture content of 13% (wet basis–w.b.).
(l) Grain yield: The total mass of grains harvested per plot was determined, and the data were converted to kg ha^−1^ at 13% (w.b.).
(m) Soil acid phosphatase: The activity of acid phosphatase in the soil was evaluated following the methodology proposed by Melo et al.^26^, which consists of incubating soil samples with the substrate sodium p-nitrophenyl phosphate and quantifying the amount of p-nitrophenol formed at the end of the incubation period.

### Statistical analysis

Initially, diagnostics for variance analysis were performed, testing the normality of the residuals and homoscedasticity using R software. The results were subjected to the F test of analysis of variance (ANOVA) for the studied factors and their interactions. When ANOVA indicated significance for the results (*p* < 0.05), polynomial regression tests were performed for the amounts of P or inoculant as isolated factors. The statistical software SISVAR^®^^27^ was used for the ANOVA and polynomial regression tests. No statistical analyses were performed to compare the crops from two years, because the experiment was conducted in different areas and with different genotypes.

Multivariate analysis was performed for the following variables: mycorrhizal fungal spores, soil acid phosphatase, root colonization, dry mass, grain productivity, mass of 1000 grains, mass of the dehusked ear, mass of grain per corncob as a function of phosphate fertilization, and AMF inoculation for 2019 and 2020. Standardization of the dataset was performed while maintaining each variable at a null mean and unit variance. Next, a principal component analysis (PCA) was performed to condense the largest amount of original information contained in n variables into p orthogonal latents called principal components (n = 8 and *p* = 2, in this case), which are linear combinations of the original variables created with the two largest eigen values of the covariance matrix of the data^28^. Thus, the set of variables was characterized by two orthogonal latent variables, called principal components (PC), enabling their representation in two-dimensional figures. The analysis was performed using data from the original variables retained by the principal components with eigen values greater than unity^29^. Analyses were conducted using STATISTICA 10.0.

### Ethical approval

All procedures with plants were conducted in accordance with the guidelines and regulation.

## Results

There was no effect of P quantities and AMF inoculation on soil acid phosphatase activity (0.00–0.20 m), with mean values of 113.22 and 37.19 mg p-nitrophenol kg^−1^ soil h^−1^ (Table 1), for 2019 and 2020, respectively. The number of AMF spores in the experimental soil was also not affected by the treatments used during the two years of the experiment, and their average values were similar (630.87 and 672.28 spores per 50 mL of dry soil for 2019 and 2020, respectively). Finally, root colonization by AMF was not altered by P fertilization or inoculation with *R. intraradices*, with averages of 61.69% in 2019 and 40.85% in 2020.

**Table 1:** Acid phosphatase activity (PHOSPHATASE), number of spores of arbuscular mycorrhizal fungi in the soil (SPORES), and percentage of mycorrhizal colonization of maize roots (COLONIZATION) as a function of applied amounts of P_2_O_5_ and inoculant containing *Rhizophagus intraradices*.

Treatment	Phosphatase		Spores		Colonization	
P_2_O_5_ (%)	mg *p*-nitrophenol kg^−1^ soil h^−1^		spores per 50 mL of dry soil		%	
	2019	2020	2019	2020	2019	2020
0.0	113.21	43.83	599.00	680.42	61.22	40.35
25.0	107.73	39.46	623.33	667.00	61.44	41.36
50.0	108.89	36.14	671.67	685.00	58.76	46.58
75.0	123.41	31.73	638.33	631.67	63.50	38.40
100.0	113.34	34.77	622.00	697.33	63.55	37.55
Inoculation (g ha^−1^)						
0.0	111.68	35.67	639.47	667.47	59.90	44.63
60.0	112.84	39.46	667.20	683.00	62.07	42.76
120.0	117.40	37.43	552.00	646.67	62.17	40.95
180.0	111.35	36.19	664.80	692.00	62.64	35.05
ANOVA (*p*-value)						
P	0.915	0.409	0.909	0.604	0.530	0.439
M	0.655	0.318	0.636	0.906	0.833	0.264
P × I	0.189	0.756	0.447	0.513	0.476	0.463
General average	113.32	37.19	630.87	672.28	61.69	40.85
CV1 (%)	39.55	40.70	30.09	15.32	14.04	29.29
CV2 (%)	12.94	15.87	43.77	26.57	16.48	33.29

*CV:* coefficient of variation (%).

In 2019, the LCI was influenced by the amount of P applied (*p* < 0.01), as shown by a decreasing linear regression equation (R^2^ = 80.55%), with an 8.22% decrease with application of maximum P compared with no application of the nutrient (Table 2 and Supplementary Fig. 3A). The mean value of this variable in 2019 was 56.82. In 2020, treatments had no effect on this variable, and the average value was higher than that in the previous year (64.12). Plant height was significantly affected by phosphate fertilization (*p* < 0.05) in 2019, as represented by an increasing linear regression equation for P doses (R^2^ = 88.34%), with a 4.48% increase at the maximum level when compared with no application of P_2_O_5_ (Supplementary Fig. 3B). In 2020, there were no effects of the analyzed factors on plant height, which were lower than those in the previous year, with an average height of 2.34 m. The corncob insertion height in 2019 was also influenced by P (*p* < 0.05), represented by a linear and increasing regression equation (R^2^ = 74.48%), and shows an increase of 5.37% at the maximum P level compared with no P_2_O_5_ addition (Supplementary Fig. 3C). In 2020, however, there was no effect of phosphate fertilization and mycorrhizal inoculation, with an average value of 1.04 m.

**Table 2:** Leaf chlorophyll index (LCI), plant height (PlantH), corncob insertion height (CorncobH), stem diameter (StemD), and shoot dry matter mass (SDM) as a function of added amounts of P_2_O_5_ and inoculant containing *Rhizophagus intraradices*.

Treatment	LCI		PlantH		CorncobH		StemD		SDM	
P_2_O_5_ (%)	–		m		m		mm		g plant^−1^	
	2019	2020	2019	2020	2019	2020	2019	2020	2019	2020
0.0	59.84^a^	61.17	2.68^b^	2.37	1.49^c^	1.05	24.16	18.58	151.53	93.37
25.0	57.30	63.54	2.73	2.32	1.53	1.01	25.00	18.42	154.62	97.61
50.0	55.70	65.89	2.78	2.32	1.56	1.01	24.73	18.49	153.45	101.27
75.0	56.36	64.37	2.78	2.34	1.54	1.07	24.17	18.79	155.27	101.27
100.0	54.92	65.64	2.80	2.31	1.57	1.05	24.12	19.03	146.17	101.20
Inoculation (g ha^−1^)										
0.0	56.42	63.39	2.74	2.33	1.52	1.04	24.59	18.74	155.32	94.31
60.0	56.41	64.20	2.76	2.31	1.55	1.03	24.20	18.55	151.62	100.57
120.0	57.46	63.99	2.75	2.36	1.53	1.03	24.89	18.61	153.31	97.78
180.0	57.00	64.91	2.75	2.33	1.54	1.04	24.05	18.74	148.59	103.12
ANOVA (*p*-value)										
P	0.007**	0.121	0.042*	0.705	0.030*	0.121	0.231	0.667	0.648	0.299
I	0.799	0.619	0.906	0.164	0.108	0.885	0.175	0.898	0.679	0.166
P × I	0.650	0.772	0.579	0.557	0.156	0.455	0.122	0.754	0.276	0.176
General Average	56.82	64.12	2.75	2.34	1.54	1.04	24.43	18.66	152.21	98.94
CV1 (%)	5.45	7.87	3.82	5.46	3.81	7.19	5.19	6.93	12.09	12.02
CV2 (%)	6.86	5.65	2.19	3.15	2.65	3.6	5.27	5.33	11.74	12.84

*CV:* coefficient of variation (%).

* and **significant at 5% and 1% by the F Test (ANOVA), respectively.

^a^y = − 0.0431x + 58.98 (R^2^ = 0.8055).

^b^y = 0.0012x + 2.696 (R^2^ = 0.8834).

^c^y = 0.0007x + 1.504 (R^2^ = 0.7448).

In both years of the experiment, there was no effect of the treatments on the diameter of the thatch, with averages of this variable in 2019 and 2020 being 24.43 mm and 18.66 mm, respectively. The shoot dry matter mass also did not show significant modifications as a function of the treatments with P and AMF, with the average values being 152.21 g plant^−1^ in 2019 and 98.24 g plant^−1^ in 2020.

Corncob length was significantly influenced by mycorrhizal inoculation in 2019 (*p* < 0.05), without the biological significance of a regression equation fit (Table 3). In 2020, there was no effect of treatment on this variable. The averages for 2019 and 2020 were 16.48 cm and 14.61 cm, respectively. In the first year of the experiment, there was an effect of inoculation with AMF on the diameter of corncobs (p < 0.05), with a fit to a quadratic regression equation (R^2^ = 72.62%) and maximum point at the applied amount of 65 g ha^−1^ of the inoculant, resulting in 5.03 diameter of diameter, an increment of 1.6% compared to no treatment (Supplementary Fig. 4A). In 2020, this variable was not affected by phosphate fertilization or inoculation with AMF, with the overall average being 4.04 cm, lower than that in 2019.

**Table 3:** Corncob length (CorncobL), corncob diameter (CorncobD), number of rows per corncob (RowsCorncob), and number of grains per row (GRows) as a function of added amounts of P_2_O_5_ and inoculant containing *Rhizophagus intraradices*.

Treatment	CorncobL		CorncobD		RowsCorncob		GRows	
P_2_O_5_ (%)	cm		cm		number		number	
	2019	2020	2019	2020	2019	2020	2019	2020
0.0	16.88	14.79	5.05	4.04	16.92	16.62	35.27	33.52
25.0	16.70	14.51	4.97	4.04	16.66	16.31	35.08	33.17
50.0	16.32	14.29	4.96	4.00	16.68	16.56	34.06	32.11
75.0	16.16	14.81	4.98	4.13	16.53	16.49	33.88	33.60
100.0	16.34	14.68	5.04	3.99	16.65	16.59	34.84	33.46
Inoculation (g ha^−1^)								
0.0	16.60	14.70	5.01^a^	4.05	16.73	16.51	34.46^b^	33.15
60.0	16.35	14.43	5.01	4.02	16.74	16.45	34.88	33.59
120.0	16.76	14.56	5.04	3.99	16.59	16.43	35.32	32.82
180.0	16.22	14.75	4.95	4.09	16.71	16.67	33.86	33.14
ANOVA (*p*-value)								
P	0.067	0.351	0.155	0.164	0.202	0.527	0.140	0.058
I	0.043*	0.259	0.044*	0.293	0.775	0.054	0.050*	0.290
P × I	0.509	0.149	0.174	0.457	0.209	0.221	0.811	0.445
General Average	16.48	14.61	5.00	4.04	16.69	16.51	34.63	33.17
CV1 (%)	4.22	5.34	2.33	3.65	2.54	3.35	4.91	4.22
CV2 (%)	3.84	3.73	2.03	3.72	3.02	1.79	4.82	3.81

*CV:* coefficient of variation (%).

*significant at 5% by the F Test (ANOVA).

^a^y = − 0.000006x^2^ + 0.000777x + 5.0062 (R^2^ = 0.7262).

^b^y = − 0.00013x^2^ + 0.02123x + 34.36400 (R^2^ = 0.8412).

Phosphate fertilization and mycorrhizal inoculation also did not affect the number of rows per corncob in the two years of the experiment, with averages of 16.69 and 16.51 rows per corncob in 2019 and 2020, respectively. The number of grains per row were benefited by inoculation with AMF (*p* < 0.05) in 2019, with quadratic regression equation fit (R^2^ = 84.12%).The maximum grains per row (35.23) were achieved after AMF application of 81.65 g·ha^−1^ (Supplementary Fig. 4B), which is 2.23% more than the mean at zero inoculation (34.46 grains per row). In 2020, this variable was not influenced by the treatments, with an overall average number being 33.17 grains per row.

Phosphate fertilization and mycorrhizal inoculation did not have significant effects on dehusked corncob mass, grain mass per ear, and cob mass. The mean of these three variables for 2019 were 162.53 g, 141.47 g, and 21.06 g, respectively; in 2020, they were 111.25 g, 95.43 g, and 15.75 g, respectively. However, there was a significant difference in the average values obtained during the two years of the experiment (Table 4).

**Table 4:** Dehusked corncob mass (DEMass), the mass of grains per corncob (MGCorncob), Cob mass (CobM), the mass of thousand grains (M1000), and grain yield (YIELD) as a function of amounts of added P_2_O_5_ and inoculant containing *Rhizophagus intraradices*.

Treatment	DEMass		MGCorncob		CobM		M1000		YIELD	
P_2_O_5_ (%)	g		g		g		g		kg ha^−1^	
	2019	2020	2019	2020	2019	2020	2019	2020	2019	2020
0.0	168.16	113.66	146.29	97.32	21.87	16.28	310.95	181.82	8210	3662
25.0	157.13	110.37	136.24	94.93	20.89	15.39	295.13	172.63	8537	4169
50.0	160.35	107.31	139.85	92.01	20.50	15.26	304.14	177.94	8220	4006
75.0	161.64	118.26	140.97	101.84	20.67	16.35	302.38	177.39	8189	4095
100.0	165.36	106.68	143.99	91.08	21.37	15.50	305.67	168.92	8793	3397
Inoculation (g ha^−1^)										
0.0	161.25	112.59	139.98	96.75	21.27	15.79	303.62	174.01	8207	4044
60.0	160.09	109.63	139.47	94.17	20.62	15.39	309.20	173.46	8192	3795
120.0	168.10	108.04	146.53	92.46	21.57	15.54	305.45	182.05	8684	3854
180.0	160.68	114.77	139.89	98.36	20.79	16.29	296.33	173.45	8476	3771
ANOVA (*p*-value)										
P	0.490	0.374	0.492	0.365	0.426	0.412	0.482	0.491	0.500	0.103
I	0.353	0.296	0.328	0.291	0.501	0.456	0.263	0.515	0.235	0.364
P × I	0.442	0.275	0.414	0.209	0.729	0.841	0.734	0.210	0.834	0.436
General average	162.53	111.25	141.47	95.43	21.06	15.75	303.65	175.74	8390	3865
CV1 (%)	11.15	15.97	11.50	16.71	10.38	12.62	6.71	11.99	11.55	21.55
CV2 (%)	9.76	10.73	9.85	10.87	10.32	11.92	5.83	12.18	8.88	13.71

*CV:* coefficient of variation (%).

Similarly, the thousand-grain mass and grain yield were not influenced by different amounts of P addition and mycorrhizal inoculation in 2019 and 2020 (Table 4). In 2019, the mean values for thousand-grain mass and yield were 303.65 g and 8390 kg ha^−1^, respectively. In 2020, the values were 175.74 g and 3865 kg ha^−1^, respectively.

Multivariate principal component analysis for the variables analyzed in 2019 (Table 5), allowed the identification of two principal components with eigenvalues greater than 1.0 (2.85 and 1.50 for components 1 and 2, respectively). The principal components (PC) allow understanding relationships between phosphate fertilization and *R. intraradices* inoculation on the variables analyzed. It was found that 54.37% of the original information was retained in the two components, 35.65% in component 1 and 18.72% in component 2. There was a joint action of the variables M1000, mass cob and grain cob for PC1. For PC2 there were higher correlation coefficients in the variables root colonization and grain yield.

**Table 5:** Eigenvalues, total and cumulative variance, eigenvectors, correlation coefficients and the two first principal components for the variables related to acid phosphatase, mycorrhizal symbiosis and the yield components in the 2019 crop.

Components	PC1	PC2
Eigenvalues	2.85	1.5
Total variance	35.65	18.72
Cumulative variance	35.65	54.37

Eigenvectors (Correlations)		
Spores (no of spores per 50 mL of dry soil)	0.18 (0.31)	− 0.31 (− 0.38)
Phosphatase (mg *p*-nitrophenol kg^−1^ soil h^−1^)	− 0.13 (− 0.22)	− 0.04 (− 0.05)
Root colonization (%)	− 0.18 (− 0.30)	− 0.65 (− 0.80)
Shoot dry matter mass (g plant^−1^)	− 0.01 (− 0.01)	− 0.25 (− 0.30)
Grain yield (kg ha^−1^)	− 0.31 (− 0.52)	0.57 (0.70)
M1000 (g)	− 0.42 (− 0.71)	− 0.30 (− 0.37)
Mass of the dehusked corncob (g)	− 0.57 (− 0.96)	0.01 (0.02)
Mass of grain per corncob (g)	− 0.57 (− 0.96)	0.02 (0.02)

*PC1* principal component one, *PC2* principal component two.

**Figure 1:**
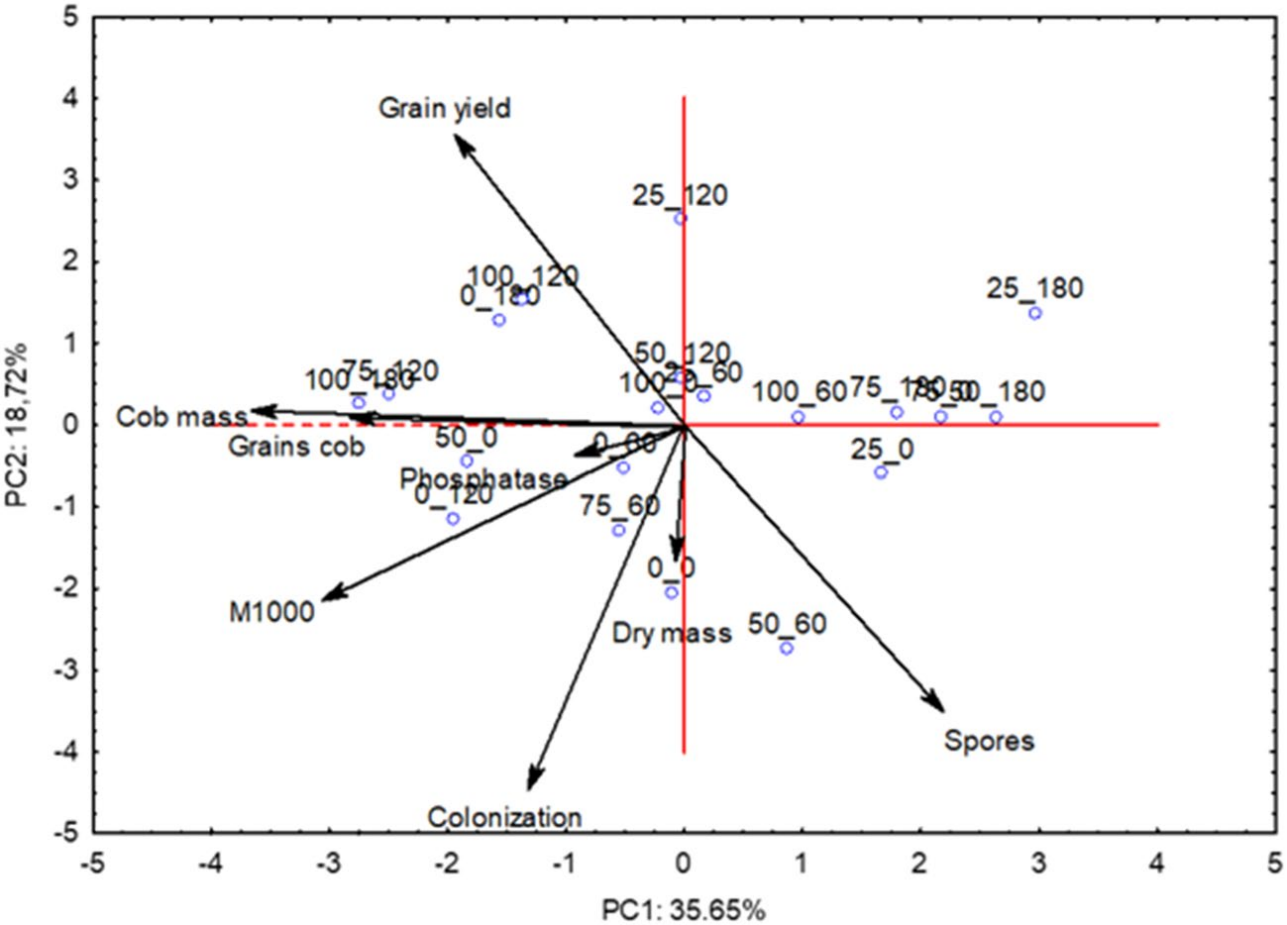
Biplot graph for the principal components PC1 and PC2 in the principal component analysis with amounts of P_2_O_5_ and inoculant containing *Rhizophagus intraradices* in 2019 season. PC1 = principal component one; PC2 = principal component two.

As shown in Figure 1, a trend towards higher grain yield in the treatments that combined 0% P_2_O_5_ with 180 g ha^−1^ inoculation, 100% P_2_O_5_ with 120 g ha^−1^ AMF, and 25% P_2_O_5_ with 120 g ha^−1^ AMF is observed. There is a negative trend in corncob mass and mass of grain per corncob in the treatments that combined 100% P_2_O_5_ with 180 g ha^−1^ of AMF, 75% P_2_O_5_ with 120 g ha^−1^ of AMF, and 50% P_2_O_5_ with 0 g ha^−1^ of AMF (no inoculation). There was a negative trend in root colonization with 75% P_2_O_5_ along with 60 g ha^−1^ of AMF, and in the absence of fertilization and inoculation (0% P_2_O_5_ and 0 g ha^−1^ of AMF).

**Figure 2:**
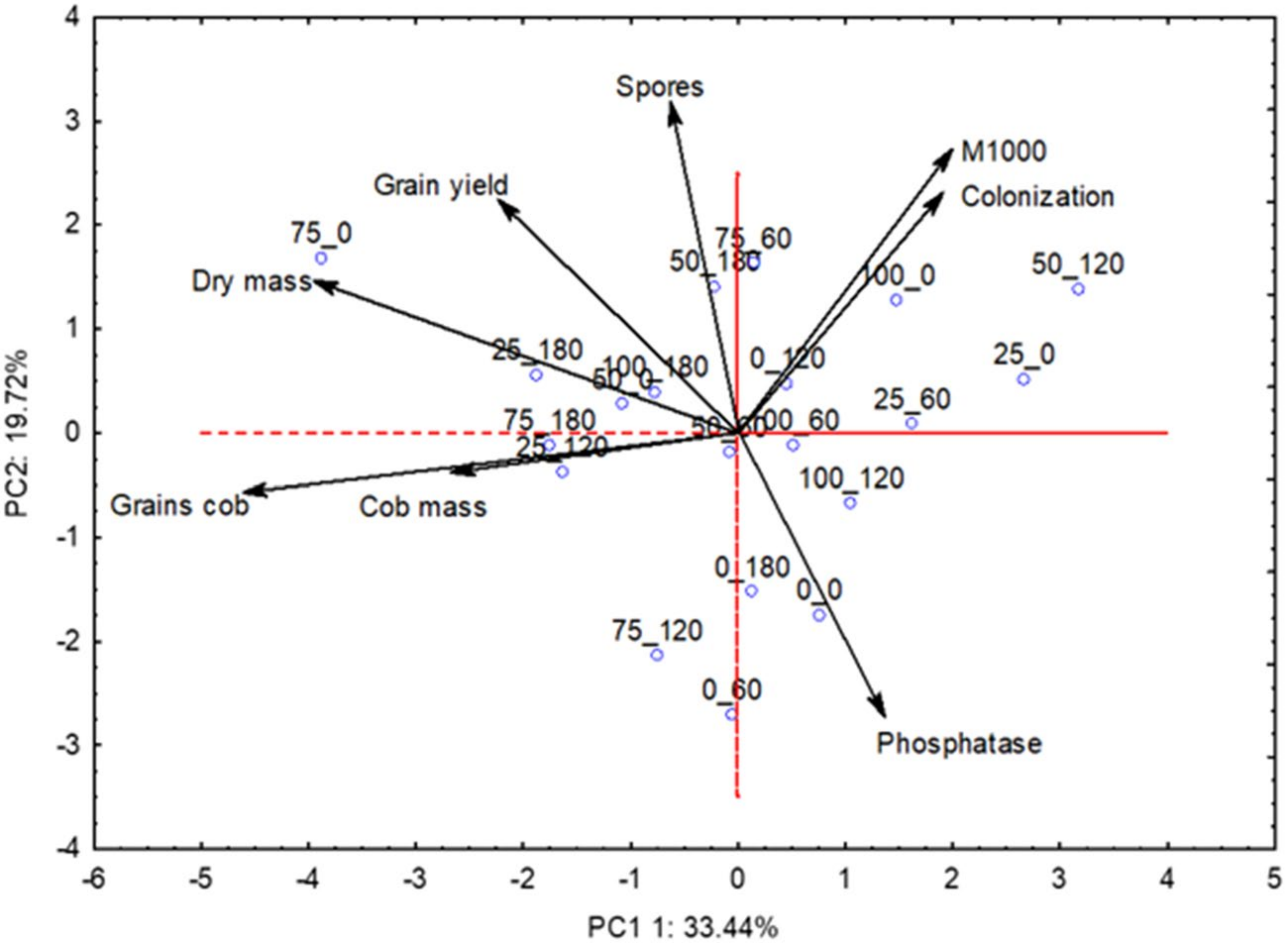
Biplot graph for the principal components PC1 and PC2 in the principal component analysis with amounts of P_2_O_5_ and inoculant containing *Rhizophagus intraradices* in 2020 season. PC1 = principal component one; PC2 = principal component two.

For multivariate analysis of the 2020 data (Table 6), we identified two PCs with eigenvalues greater than 1 (2.67 and 1.58 for components 1 and 2, respectively). The two components retained 53.16% of their original information, with 33.44% in PC1 and 19.72% in PC2. There was a joint action of the variables corncob mass and mass of grain per corncob for PC1 and mycorrhizal fungal spores for PC2. Figure 2 indicates that there is a negative trend of corncob mass and mass of grain per corncob in the treatments that combined 75% P_2_O_5_ with 180 g ha^−1^ of AMF, and 25% P_2_O_5_ with 120 g ha^−1^ of AMF, similar to what occurred in 2019. A positive trend of mycorrhizal fungal spores in the soil is observed after treatments with 50% P_2_O_5_, 180 g ha^−1^ AMF, 75% P_2_O_5_, and 60 g ha^−1^ AMF.

**Table 6:** Eigenvalues, total and cumulative variance, eigenvectors, correlation coefficients and the two first principal components for the variables related to acid phosphatase, mycorrhizal symbiosis and the yield components in the 2020 crop.

Components	PC1	PC2
Eigenvalues	2.67	1.58
Total variance	33.44	19.72
Cumulative variance	33.44	53.16

Eigenvectors (Correlations)		
Spores (no of spores per 50 mL of dry soil)	− 0.10 (− 0.17)	0.63 (0.79)
Phosphatase (mg *p*-nitrophenol kg^−1^ soil h^−1^)	0.20 (0.33)	− 0.48 (− 0.61)
Root colonization (%)	0.23 (0.38)	0.32 (0.40)
Shoot dry matter mass (g plant^−1^)	− 0.39 (− 0.63)	0.16 (0.20)
Grain yield (kg ha^−1^)	− 0.21 (− 0.34)	0.24 (0.29)
M1000 (g)	0,26 (0.42)	0.41 (0.52)
Mass of the dehusked corncob (g)	− 0,56 (− 0.92)	− 0.08 (− 0.10)
Mass of grain per corncob (g)	− 0.56 (− 0.92)	− 0.08 (− 0.11)

*PC1* principal component one, *PC2* principal component two.

## Discussion

This study investigated the effects of phosphate fertilization and inoculation of arbuscular mycorrhizal fungi in maize crop. The activity of the enzyme acid phosphatase in the soil showed values within the range commonly found for the activity of this enzyme in soil^30,31^. It has already been shown in literature that higher soil P availability can inhibit the activity of acid phosphatase due to the metabolic expenditure involved in the production of the enzyme^32^, which is different from what was found in the present work. This occurred because in maize plants, the effect of fertilizer P addition on this enzyme depends on the genotype and its susceptibility to soil P deficiency^33^. Moreover, this enzyme is involved in the mineralization of organic P to inorganic P in the soil, and its activity depends more on the stock of organic material in the soil than on the addition of mineral fertilizers^34^. This explains the lack of differences in the activity of acid phosphatase as a function of phosphate fertilization in the two years of this study.

Although symbiosis with AMF has the potential to increase phosphatase activity in the soil^35,36^, this does not always occur, as it was found in the present work, because the most important contribution of these fungi to plant phosphate nutrition is through the uptake and transport of phosphate between the soil and the plant^37^.

In the two years of our experiment, the number of spores obtained in the soil was higher than that reported in the literature^38,39^. This was due to the no-till system and the use of millet in the area, because the lack of soil disturbance and the use of cover crops kept populations of native AMF high^40,41^.

The large number of spores detected in inoculated and non-inoculated plots may be due to the presence of highly sporulating native AMF commonly found in agricultural soils, including isolates of R. irregularis. Several isolates of this species are adapted to conditions of high fertilization and soil disturbance^42–44^ and are known to produce many spores and extra-radical mycelium^45,46^.

In this regard, the high number of spores obtained in the soil resulted in high root colonization of maize plants (61.69% for 2019 and 40.85% for 2020) regardless of inoculation. Previous studies have reported lower values for root colonization in maize, with values less than 56%^47^ and minimum of 39.3%^41^ and 22.0%^48^. Therefore, the practice of seed inoculation did not increase the shoot dry matter mass of maize plants. Symbiosis with AMF, can promote greater vegetative development of plants by providing greater water and nutrient uptake^8,49,50^, however, in soils with a higher native population of these fungi, the practice of inoculation may not be necessary to achieve these effects.

The decrease in LCI with increasing P supply in 2019 can be explained by the lower chlorophyll concentration in plants that received higher phosphate fertilization (dilution effect). When P is limited, leaves are darker green due to the high chlorophyll content per unit area, because vegetative leaf growth is slower than chlorophyll formation in these plants. Thus, this lower LCI as a function of the higher P_2_O_5_ doses is not harmful, because with the lower P supply, the photosynthetic efficiency is lower^51,52^.

The increase in maize plant height as a function of phosphate fertilization were also verified by Fiorini et al.^53^. The increase in maize height with P_2_O_5_ doses occurred because P is essential for photosynthesis, respiration, storage, and transfer of energy (and in cell division and growth and is present in plant compounds such as phosphate sugars, phospholipids of cell membranes, coenzymes, and nucleotides^54,55^.

In this work, the amount of P already available in the soil (16 and 25 mg dm^−3^ for 2019 and 2020, respectively), added to that supplied by the mycorrhizal symbiosis was sufficient to satisfy the needs of the plants for this nutrient, resulting in no difference in production components and yield as a function of phosphate fertilization even with reduced or zero amounts of added P_2_O_5_.

The association of AMF with plants has the potential to improve crop production, as evidenced by the benefit of inoculation on corncob diameter and the number of grains per row in 2019 and by studies verifying increases in dry plant biomass and grain yield in annual crops and tree species due to mycorrhizae^50,56,57^. However, due to the high presence of native spores in the soil and the average available P content, these results were not evident in shoot dry matter mass, thousand kernel mass and yield.

Furthermore, well-managed soils, with cover crops, no-tillage, and rational fertilization provide a favorable environment for AMF to establish efficient symbiotic relationships with plants, and inoculation is not necessary^58^. Thus, inoculation may only have significant effects when soils are limited in terms of AMF propagules, i.e. soils with a lower amount of native AMF spores than found in the present work.

We observed that in the second year of the experiment, the mean values found for the variables were lower than the mean values of 2019, resulting in the mean yield of 2020 being more than 50% lower than that of 2019. This was due to an unexpected failure of the hybrid used in 2020 to adapt to the high temperatures of the region. However, this did not cause any problems in the analysis of the treatments for the development and yield of the crop.

The multivariate analysis confirmed what was seen with the ANOVA and regression analyzes. In 2019, the yield result indicated that inoculation with *R. intraradices* can increase maize yield and allow a better utilization of applied P_2_O_5_, as shown in the literature^5,7^. In the same year, for maize corncob mass and grain mass per corncob, the negative trend of the multivariate shows that reducing P_2_O_5_ while not inoculating AMF, can harm maize yield components, this is because phosphorus is essential for plants as an ATP molecule component, participates in photosynthesis and respiration and is part of sugar molecules, membrane phospholipids and coenzymes^45^ and with less inoculation, there may be less P uptake. The negative trend in maize corncob mass and grain mass per corncob in the treatments combining100% P_2_O_5_ with 180 g ha^−1^ AMF and 75% P_2_O_5_ with 120 g ha^−1^ in 2019 and in the treatments combining 75% P_2_O_5_ with 180 g ha^−1^ AMF in 2020 is due to thefact that the plant will provide photoassimilates to the AMF in symbiosis, but will not receive a proportional return because there is sufficient phosphorus available in the soil^59^. In other words, it is likely that AMF inoculation will need to be adjusted to the applied P_2_O_5_ in order for the plant to take advantage of the applied symbiosis and P_2_O_5_ without over-fertilizing or spending photoassimilates with symbiotic AMF that are not returned in proportional benefits to the plant.

The negative trend of root colonization in 2019 at higher doses of P_2_O_5_ and lower doses of inoculation is due to the fact that high doses of phosphate fertilization can inhibit root colonization by AMF^60,61^ and demonstrates that inoculation can increase colonization in maize. The positive trend in mycorrhizal spores seen in 2020 indicates that inoculation of *R. intraradices* can increase the amount of AMF spores in the soil, resulting in an increased native population for mycorrhiza development of subsequent crops.

## Conclusions

This study concluded that inoculation of *R. intraradices* in maize crops has the potential to improve crop development and yield, depending on the factors involved in symbiosis. Furthermore, under conditions of high colonization of plants by native AMF species and availability of phosphorus in the soil, there was no immediate effect of inoculation on the efficiency of phosphate fertilization. Finally, phosphate fertilization is essential for the development and yield of maize, but its immediate effects may not be observed in soils with good fertility.

## Supplementary Information

Supplementary Figure 1.

Supplementary Figure 2.

Supplementary Figure 3.

Supplementary Figure 4.

## Data Availability

The datasets used and analyzed during the current study available from the corresponding author on reasonable request.
