# Supplementary figures and images for: Effect of mycorrhizae on phosphate fertilization efficiency and maize growth under field conditions
Source: Sci Rep. 2023 Mar 2;13:3527. doi: 10.1038/s41598-023-30128-7 (PMC9981755; doi:10.1038/s41598-023-30128-7)

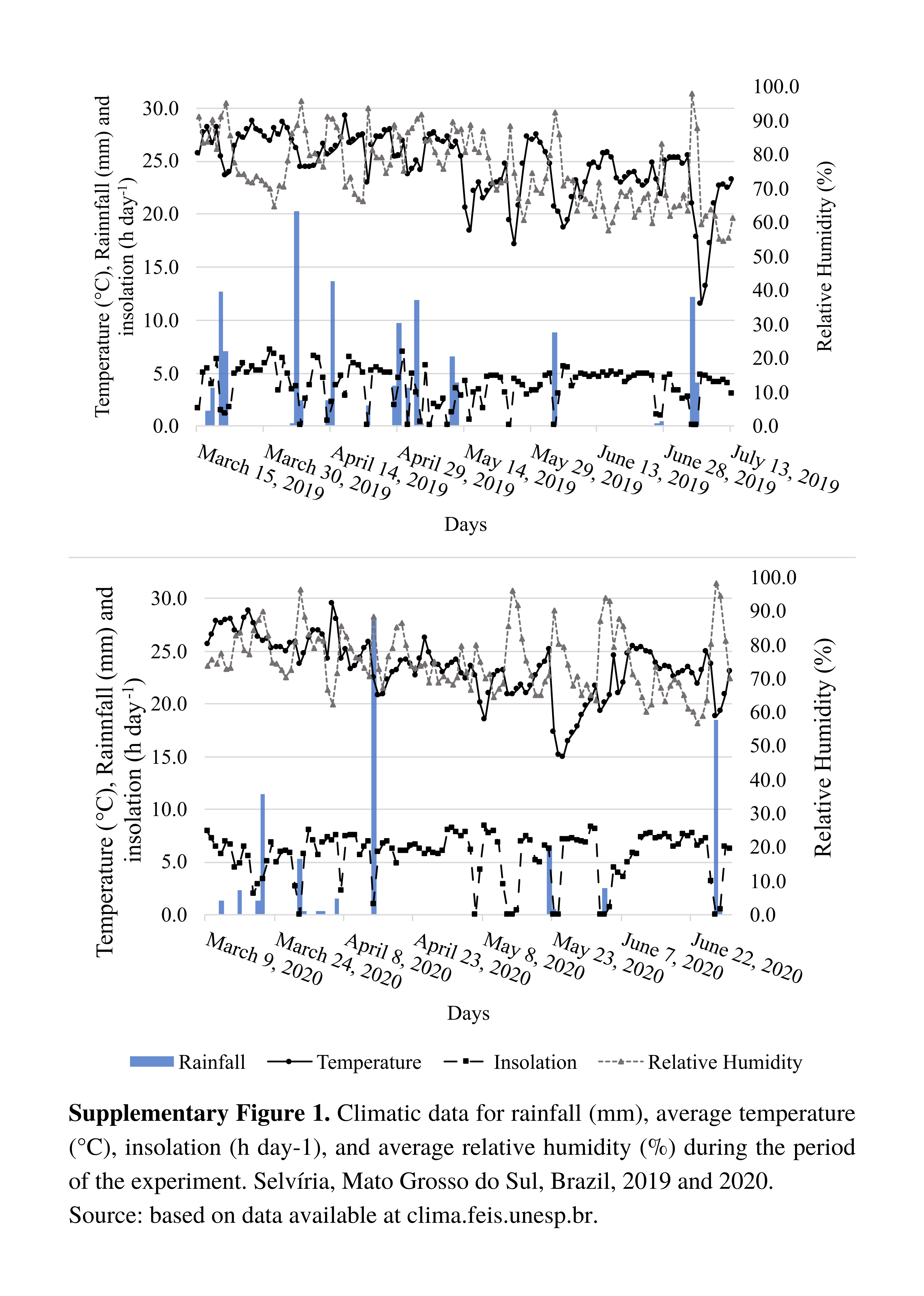

Supplement: Supplementary file 1 — Supplementary Figure 1. [file 41598_2023_30128_MOESM1_ESM.png]

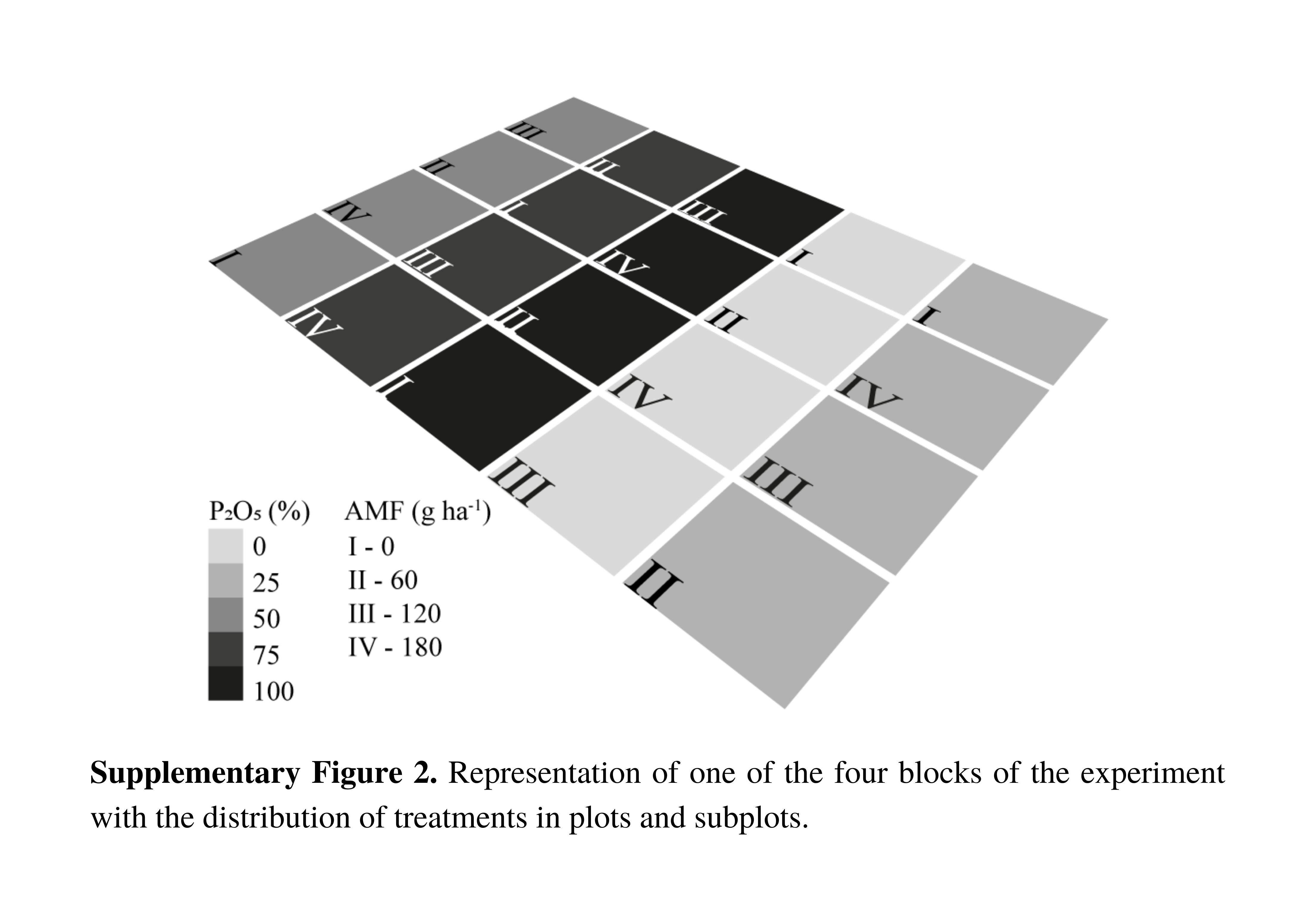

Supplement: Supplementary file 2 — Supplementary Figure 2. [file 41598_2023_30128_MOESM2_ESM.png]

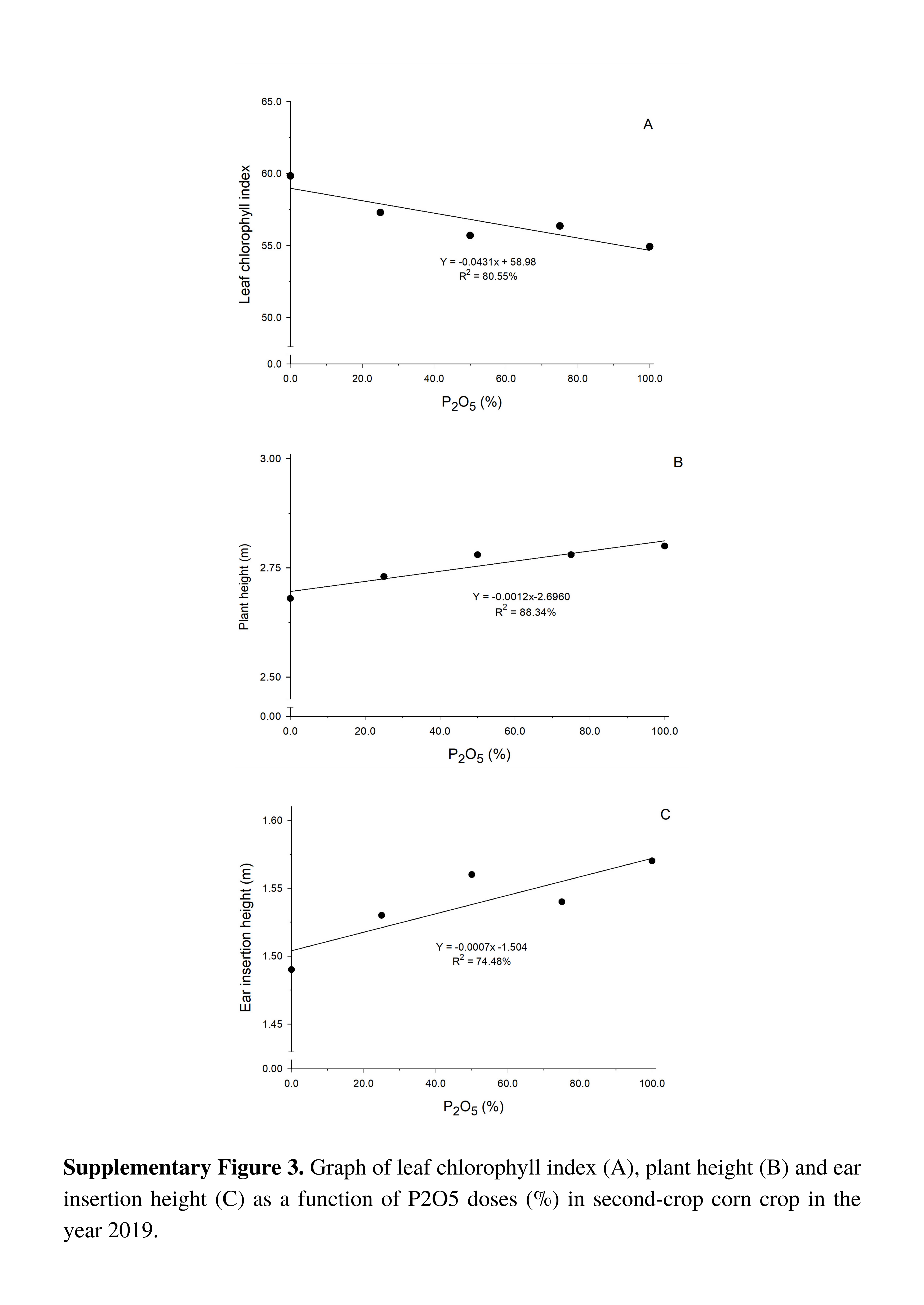

Supplement: Supplementary file 3 — Supplementary Figure 3. [file 41598_2023_30128_MOESM3_ESM.png]

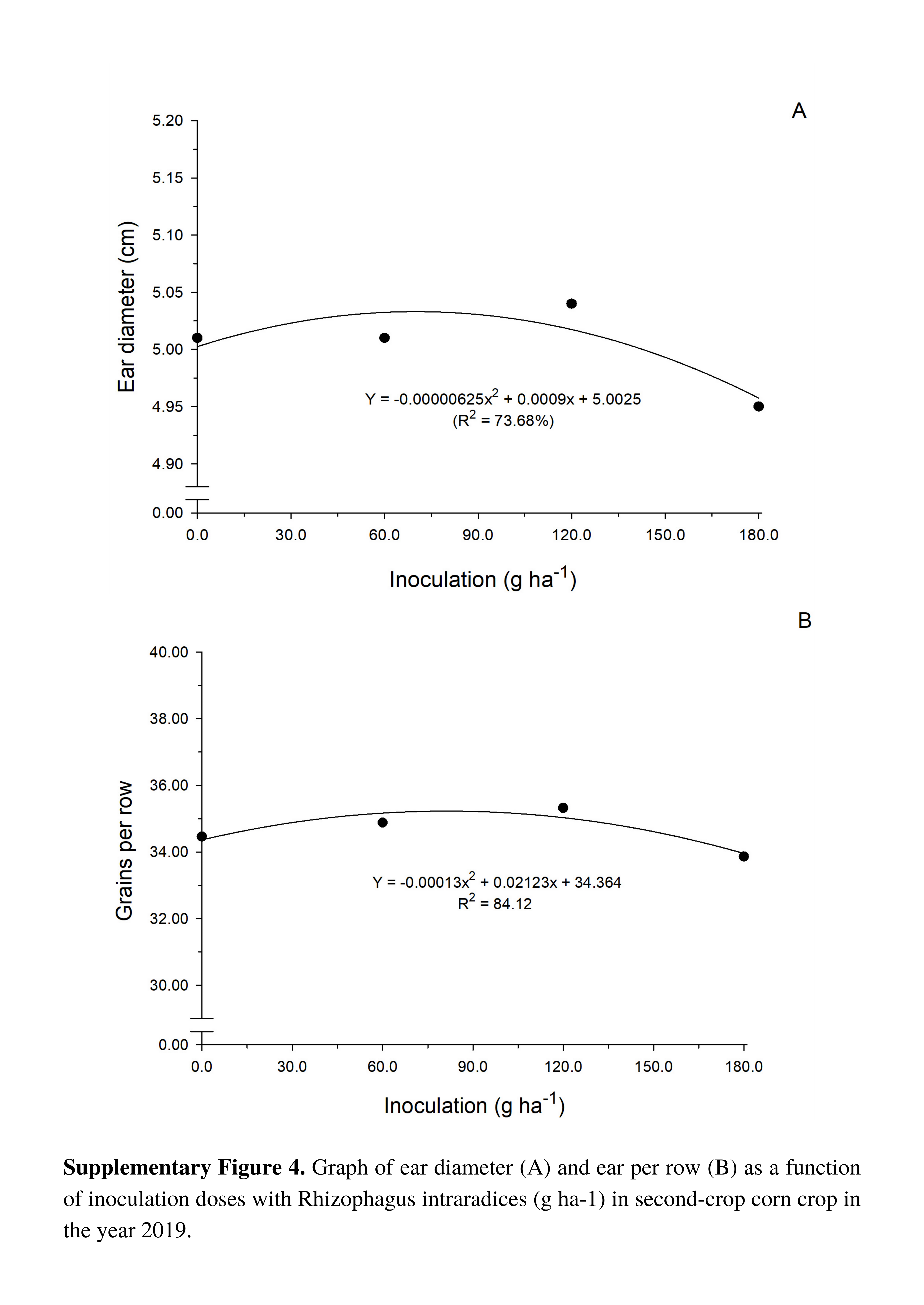

Supplement: Supplementary file 4 — Supplementary Figure 4. [file 41598_2023_30128_MOESM4_ESM.png]
